# Reactivación de la enfermedad de Chagas después de trasplante autólogo de progenitores hematopoyéticos. Reporte de caso y revisión de la literatura

**DOI:** 10.7705/biomedica.6288

**Published:** 2022-06-01

**Authors:** Juan David Rojas, Mario Pereira, Bibiana Martínez, Julio César Gómez, Sonia Isabel Cuervo

**Affiliations:** 1 Departamento de Medicina Interna, Facultad de Medicina, Universidad Nacional de Colombia, Bogotá, D.C., Colombia Universidad Nacional de Colombia Universidad Nacional de Colombia Bogotá D.C. Colombia; 2 Grupo de Investigación en Enfermedades Infecciosas en Cáncer y Alteraciones Hematológicas, Bogotá, D.C., Colombia Grupo de Investigación en Enfermedades Infecciosas en Cáncer y Alteraciones Hematológicas Bogotá D.C. Colombia; 3 Grupo de Trasplante de Precursores Hematopoyéticos, Instituto Nacional de Cancerología E.S.E., Bogotá, D.C., Colombia Instituto Nacional de Cancerología E.S.E Bogotá D.C. Colombia; 4 Grupo de Patología, Instituto Nacional de Cancerología E.S.E., Bogotá, D.C., Colombia Instituto Nacional de Cancerología E.S.E Bogotá D.C. Colombia; 5 Grupo de Infectología, Instituto Nacional de Cancerología E.S.E., Bogotá, D.C., Colombia Instituto Nacional de Cancerología E.S.E Bogotá D.C Colombia

**Keywords:** Trypanosoma cruzi, enfermedad de Chagas, mieloma múltiple, trasplante de células madre hematopoyéticas, neutropenia febril, enfermedades parasitarias, inmunosupresión, Trypanosoma cruzi, Chagas disease, multiple myeloma, hematopoietic stem cell transplantation, febrile neutropenia, parasitic diseases, immunosuppression

## Abstract

**Introducción.:**

La enfermedad de Chagas es una parasitosis endémica en Latinoamérica transmitida por triatominos. Está asociada a factores de riesgo como la pobreza y la ruralidad. Después de la infección aguda, un tercio de los pacientes presenta compromiso del corazón, el aparato digestivo o el sistema nervioso central, en tanto que los dos tercios restantes no presentan este tipo de compromiso secundario. La inmunosupresión farmacológica rompe el equilibrio entre el sistema inmunitario y el parásito, lo cual favorece su reactivación.

**Caso clínico.:**

Se presenta el caso de un hombre de 58 años procedente de un área rural colombiana, con diagnóstico de mieloma múltiple resistente a los fármacos de primera línea de tratamiento, que requirió un nuevo esquema de quimioterapia y consolidación con trasplante autólogo de células madre.

Después del trasplante, presentó neutropenia febril. Los estudios microbiológicos iniciales fueron negativos. En el frotis de sangre periférica, se demostraron tripomastigotes y se diagnosticó enfermedad de Chagas aguda posterior al trasplante. Se inició el tratamiento con benznidazol. La evolución del paciente fue satisfactoria.

**Conclusiones.:**

La serología positiva para Chagas previa a un trasplante obliga a descartar la reactivación de la enfermedad en caso de neutropenia febril. Se requieren más estudios para determinar las herramientas que permitan estimar la probabilidad de reactivación de la enfermedad y decidir sobre la mejor opción de relación entre costo, riesgo y beneficio de la terapia profiláctica.

## Abstract (en)

**Introduction:**

Chagas disease is an endemic parasitic infection in Latin America transmitted by triatomines. It is associated with risk factors such as poverty and rurality. After acute infection, a third of patients will present target organ involvement (heart, digestive tract, central nervous system). The remaining two thirds remain asymptomatic throughout their life. Pharmacological immunosuppression breaks the balance between the immune system and the parasite, favoring its reactivation.

**Clinical case:**

We present the case of a 58-year-old man from a Colombian rural area with a diagnosis of multiple myeloma refractory to the first line of treatment who required a new chemotherapy scheme and consolidation with autologous stem cell transplant. During the post-transplant period, he suffered from febrile neutropenia. Initial microbiological studies were negative but the peripheral blood smear evidenced trypomastigotes in blood. With a diagnosis of acute Chagas disease in a post-transplant patient, benznidazole was started. The evolution of the patient was satisfactory.

**Conclusions:**

Positive serology prior to transplantation makes it necessary to rule out reactivation of the pathology in the setting of febrile neutropenia. More studies are required to determine tools for estimating the probability of reactivation of the disease and defining the best cost-risk-benefit relation for the prophylactic therapy.

La enfermedad de Chagas es causada por *Trypanosoma cruzi*, un hemoparásito protozoario flagelado transmitido mediante inoculación de heces infectadas de triatominos y, menos frecuentemente, mediante transfusión sanguínea, trasplante de órgano sólido, transmisión oral o por vía transplacentaria ^1^^,^^2^. Está asociada a factores de riesgo como la pobreza o la ruralidad y es endémica en Latinoamérica. Después de la infección aguda, un tercio de los pacientes presenta compromiso de algún órgano blanco (corazón, aparato digestivo, sistema nervioso central) ^3^. Los dos tercios restantes no presentan este tipo de compromiso secundario a la infección primaria. La inmunosupresión farmacológica rompe el equilibrio entre el sistema inmunitario y el parásito, lo cual favorece su reactivación ^4^.

Se presenta el caso de un hombre de 58 años procedente de un área rural colombiana, con diagnóstico de mieloma múltiple resistente a los fármacos de primera línea de tratamiento, quien cursó con neutropenia febril secundaria a enfermedad de Chagas aguda después del trasplante.

## Caso clínico

Se trata de un hombre de 58 años, natural de Guamo (Tolima) y procedente de Acacías (Meta), con mieloma múltiple secretor de cadena ligera lambda, diagnosticado en enero de 2018 (inmunofenotipo: CD38, CD 138, CD 56 y CD117 positivo; CD45 y CD 19 negativo) y el 58 % de células plasmáticas en médula ósea; el componente CRAB se había expresado en fracturas en T8, T10 y T12 y anemia (6,6 g/dl).

Recibió seis ciclos de tratamiento con bortezomib, talidomida, dexametasona (protocolo VTD) más ácido zolendrónico. Presentó una recaída en agosto del 2019 con 16 % de plasmocitos en médula ósea. Recibió el protocolo con carfilzomib, lenalidomida, dexametasona (protocolo KRD) en 8 ciclos hasta el 21 de mayo del 2020.

Durante la valoración previa al trasplante autólogo, el ensayo de inmunoabsorción ligado a enzima fue positivo para Chagas. Se estableció que, durante su infancia, el paciente había vivido en una casa de bahareque y techo de paja y sufrió “picaduras de pitos”. En la revisión por sistemas, no se reportaron síntomas de origen cardiaco (disnea, palpitaciones, sincope) ni gastrointestinal (disfagia, estreñimiento).

En el examen físico, el estado general era aceptable, el paciente estaba alerta, orientado y con los siguientes signos vitales: presión arterial, 133/76 mm Hg; frecuencia cardíaca, 72 latidos por minuto; frecuencia respiratoria, 12 por minuto; peso, 72 kg; talla, 168 cm; IMC, 25,51; e índice de Karnofsky, 80 puntos. No hubo hallazgos anormales cardiovasculares ni abdominales y el examen neurológico no evidenció signos focales. Los estudios inmunológicos antes del trasplante evidenciaron infección por *T. cruzi* (cuadro 1).

**Cuadro 1:** Estudios inmunológicos previos al trasplante

Microorganismo		Valor	Interpretación
Citomegalovirus	IgM CMV	19,6 R	Infección previa
	IgG CMV	0,34 NR	
Virus herpes simple I	IgM HSV I	0,33 NR	Infección previa
	IgG HSV I	1,21 R	
Virus herpes simple II	IgM HSV II	0,04 NR	Sin infección
	IgG HSV II	0,8 NR	
*Toxoplasma gondii*	IgM	0,31 NR	Infección previa
	IgG	295 R	
Virus de la hepatitis B	HBAgS	0,492 NR	Sin infección
	HBAcS	<2,0 NR	
	ANTI-HBC	2,31 NR	
Virus de las hepatitis A y C	ANTI-VHA	0,02 NR	
	ANTI-VHC	0,03 NR	
Retrovirus	ANTI-HTLV-I/II	0,11 NR	
	HIV	0,208 NR	
*Mycobacterium tuberculosis*	Prueba de Mantoux	3 mm	Negativo
*Trypanosoma cruzi*	Inmunofluorescencia indirecta	1:256	Infección

R: reactivo; NR: no reactivo

Con base en dichos resultados, se diagnosticó infección por Chagas sin manifestación de enfermedad activa en órganos blanco como el corazón, ni en el sistema mononuclear fagocítico o el nervioso central, por lo que se acordó hacer el trasplante. Se propuso hacer un frotis de sangre periférica al no tener acceso a una PCR ni a la estandarización por el método de Strout para detectar el parásito desde el inicio del régimen de acondicionamiento, dado que la parasitemia predice efectivamente la reactivación de la enfermedad de Chagas en esta situación y no había evidencia suficiente para recomendar el tratamiento anticipado.

**Figura 1:**
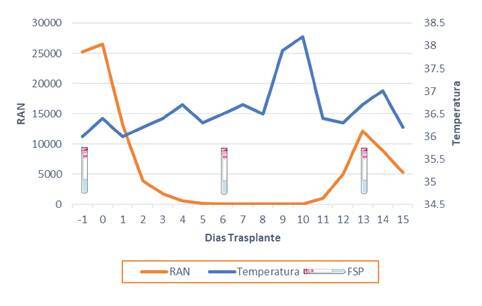
Relación entre temperatura axilar, recuento absoluto de neutrófilos (RAN) y cronología de los frotis de sangre periférica

El paciente ingresó en octubre para el trasplante autólogo de progenitores hematopoyéticos (cuadro 2). A los diez días del trasplante, el paciente cursaba con neutropenia febril (figura 1), por lo que se inició el tratamiento con cefepime y vancomicina. No había alteraciones en las imágenes de la región del tórax. Se determinó hacer semanalmente frotis de sangre periférica para detectar la parasitemia durante los dos primeros meses del trasplante; posteriormente, se decidió realizarlo cada dos semanas durante el tercer mes y, luego, mensualmente durante un período que sería determinado por la situación clínica específica (inmunosupresión intensificada, fiebre inexplicable, enfermedad de injerto contra huésped).

**Cuadro 2:** Cronología de intervenciones diagnósticas y terapéuticas

Día del trasplante	Evento	Aproximación al diagnóstico	Tratamiento
-6 a -2	Movilización con plerixafor; factor estimulante de colonias granulocíticas	NA	NA
-1	Acondicionamiento con melfalán	Frotis de sangre periférica	
0	Infusión de progenitores hematopoyéticos		
+3			Dexametasona
+6		Frotis de sangre periférica	Filgrastim
+10	Neutropenia febril, único episodio	Hemocultivos (tres), galactomanano Cefepime y vancomicina sérico	
+11	Injerto granulocítico	Radiografía de tórax, PCR-SARS- CoV-2	Suspensión de dexametasona
+12		Tomografía computarizada de tórax	
+13	Tripomastigotes	Frotis de sangre periférica	Benznidazol
+15	Envío lámina a Instituto Nacional de Salud	Ecocardiograma transtorácico	Suspensión de antibióticos

NA: no aplica

Durante su estancia hospitalaria, el paciente permaneció asintomático, con excepción de un único pico febril en el día 10 del trasplante. En el examen físico se registró una única elevación de la temperatura axilar (8 noviembre de 2020) de 38,2 °C. El paciente presentaba un aceptable estado general, se mostraba consciente y alerta, y no hubo nuevos hallazgos cardiovasculares ni gastrointestinales. Los hemocultivos, el urocultivo, el galactomanano sérico y la prueba para SARS-CoV-2, fueron negativos.

**Figura 2:**
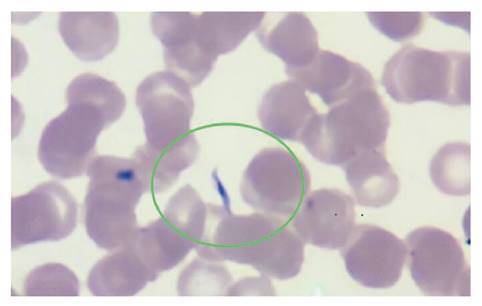
Extendido de sangre periférica, 40X. Se observan eritrocitos sin alteración de la morfología; no se observan leucocitos ni plaquetas. En la región demarcada, se evidencia un tripomastigote.

En el frotis de sangre periférica del día 13 después del trasplante, se detectaron formas parasitarias correspondientes a *Trypanosoma* (figura 2). Al día siguiente, se enviaron las láminas con el extendido al Instituto Nacional de Salud donde un segundo observador confirmó el hallazgo.

El reporte del ecocardiograma evidenció remodelación concéntrica del ventrículo izquierdo y función sistólica conservada (fracción de eyección del ventrículo izquierdo-FEVI: 61 %), disfunción diastólica de tipo 1, cavidades derechas normales, insuficiencia tricúspide trivial, baja probabilidad de hipertensión pulmonar y pericardio normal. Dado el diagnóstico de enfermedad aguda de Chagas, después de trasplante se inició la administración de benznidazol por siete semanas.

El paciente fue dado de alta en el día 16 después del trasplante dada su adecuada evolución clínica y paraclínica, con la indicación de hacer un frotis de sangre periférica semanalmente hasta la finalización del tratamiento. Recibió 60 días de tratamiento oral con benznidazol sin efectos secundarios asociados. Debido a su procedencia lejana, no fue posible hacer ambulatoriamente el frotis de sangre periférica. En el control ambulatorio institucional tres meses después del trasplante, no presentaba recurrencia de los síntomas y, ante la ausencia de un régimen de inmunosupresión intensificado, o nuevos episodios de neutropenia o fiebre inexplicable, se consideró que no había necesidad de nuevos frotis de sangre periférica.

A los 16 meses después del trasplante, el paciente estaba estable y se le mantenían los controles ambulatorios.

## Consideraciones éticas

Los autores confirman que el consentimiento informado por escrito para la presentación y publicación de este informe del caso, incluidas las imágenes y el texto asociado, se obtuvieron del paciente de acuerdo con la guía del *Committee on Publication Ethics* (COPE).

## Discusión

El mieloma múltiple es una neoplasia de células plasmáticas cuyo diagnóstico requiere la presencia de uno o más eventos definitorios de mieloma asociados al 10 % o más de células plasmáticas clonales en médula ósea. La elección del tratamiento inicial depende de la estratificación del riesgo y la elegibilidad para el trasplante autólogo ^5^. El trasplante autólogo puede ser temprano o tardío (posterior a la primera recaída), dado que no hay diferencia en la supervivencia global ^6^. Nuestro paciente presentaba el riesgo estándar y era candidato a trasplante autólogo; tuvo una recaída después de recibir seis ciclos de VTD, tras lo cual se propuso la terapia de inducción con KRD y trasplante autólogo. El trasplante alogénico en los casos de mieloma múltiple exige someter al paciente a un mayor tiempo de inmunosupresión.

La enfermedad de Chagas es endémica en 21 países de América, con seis millones de personas infectadas. Presenta una incidencia anual de 30.000 nuevos casos en promedio y una mortalidad estimada de 12.000 personas por año ^2^. La causa de la enfermedad es *T. cruzi*, un protozoo hemoflagelado transmitido por inoculación de heces infectadas de triatominos y, menos frecuentemente, por transfusión sanguínea, trasplante de órgano sólido, por vía oral y por vía transplacentaria ^1^. La progresión natural de la enfermedad implica una fase aguda sintomática, en la mayoría de los casos no diagnosticada, seguida de una fase asintomática o indeterminada que puede durar entre 10 y 30 años, o incluso toda la vida del huésped. La progresión a la fase final crónica ocurre en un tercio de todas las infecciones ^7^.

En la fase aguda de la enfermedad de Chagas, se registran altos niveles de parasitemia e invasión tisular sistémica. Cursa como una enfermedad febril aguda con linfadenomegalias y hepatoesplenomegalia, que se resuelve espontáneamente, incluso sin tratamiento específico ^8^. La infección persiste a pesar de la evidencia serológica de inmunidad, lo que, en ausencia de síntomas, configura la fase indeterminada, periodo de mayor propensión a la reactivación, dado que la ausencia de síntomas limita el seguimiento de la enfermedad ^7^. En la fase crónica, la parasitemia es solo detectable por métodos indirectos como el hemocultivo o el xenodiagnóstico, y se caracteriza por el compromiso secundario cardiaco (27 %), gastrointestinal (6 %) y del sistema nervioso periférico (3 %) ^7^.

El principal factor de riesgo para su reactivación es la inmunosupresión farmacológica, la cual rompe el equilibrio entre la reacción inmunológica y el parásito. No se ha establecido aún cuál factor contribuye más a la reactivación, si la inmunosupresión inducida por la quimioterapia o la enfermedad *per se*, o el trasplante autólogo de precursores hematopoyéticos ^9^. Durante la reactivación, las manifestaciones clínicas incluyen síndrome febril agudo, miocarditis, meningoencefalitis y lesiones cutáneas ^7^^,^^10^.

El diagnóstico en las fases indeterminada y crónica se hace mediante la detección de anticuerpos de los antígenos de *T. cruzi*, cuyo rendimiento diagnóstico depende de la calidad del antígeno y el tipo de prueba utilizada ^11^. Las más comunes son la prueba de inmunoabsorción ligada a enzimas (ELISA), la inmunofluorescencia indirecta y la hemaglutinación. Dado su bajo rendimiento, se requieren dos pruebas diferentes para el diagnóstico y, en caso de discordancia, una tercera ^7^^,^^11^. Cuando hay infección aguda o reactivación, los exámenes serológicos carecen de utilidad, y debe recurrirse a la microscopía directa o la amplificación de ácidos nucleicos mediante PCR ^8^^,^^11^.

Dada la inmunosupresión grave que conlleva el trasplante autólogo, se hacen la evaluación clínica y las tamizaciones infecciosas requeridas antes de proceder ^12^. Las enfermedades parasitarias son las menos estudiadas de todas las infecciones relacionadas con el trasplante autólogo ^8^. En Colombia, se hace tamización para *T. cruzi* por ser un país endémico. No hay una prueba estándar para el diagnóstico serológico, por lo que se sugiere hacer dos pruebas serológicas distintas ^12^. En nuestro caso, el paciente había vivido en áreas endémicas toda su vida y casi siempre en condiciones de alto riesgo. Aunque la seropositividad no es una contraindicación para el trasplante, es un diagnóstico diferencial que se debe tener en cuenta en caso de enfermedad aguda, particularmente durante la neutropenia ^12^.

La evidencia clínica disponible no ha permitido emitir recomendaciones sobre la terapia profiláctica o preventiva para el trasplante autólogo, aunque en reportes iniciales algunos expertos han recomendado benznidazol o nifurtimox. Dictar, *et al*., reportaron por primera vez una serie de casos de cinco pacientes con enfermedad de Chagas crónica que fueron sometidos a trasplante de médula ósea ^13^ (cuadro 3). Un paciente con leucemia mieloide aguda y otro con leucemia mieloide crónica, fueron sometidos a trasplante alogénico, y dos pacientes con linfoma no Hodgkin y uno con leucemia linfoide aguda, a trasplante autólogo. En estos últimos tres casos, se utilizó el método de Strout y se hicieron hemocultivos, una prueba ELISA, inmunofluorescencia indirecta y hemaglutinación indirecta semanalmente, desde el inicio de la quimioterapia hasta el día 60 después del trasplante. Ningún paciente recibió profilaxis. Se detectaron tripomastigotes en un hemocultivo de uno de los pacientes con trasplante autólogo de médula ósea durante su periodo de granulocitopenia, el cual no presentaba síntomas. Se inició el tratamiento preventivo antes de la infusión de la medula ósea con benznidazol durante 30 días, con lo que hubo un descenso progresivo de la parasitemia, con xenodiagnóstico negativo al finalizar la terapia.

**Cuadro 3:** Diagnóstico y manejo de la reactivación de la enfermedad de Chagas

Autor	Población y exámenes paraclínicos	Hallazgos	Tratamiento
Dictar, *et al*., 1998 ^13^	5 pacientes con enfermedad de Chagas crónica sometidos a trasplante de médula ósea Strout, hemocultivos, ELISA, inmunofluorescencia indirecta y hemaglutinación semanalmente desde el inicio de la quimioterapia hasta el día 60 del trasplante	1 paciente asintomático con hemocultivo positivo	Tratamiento preventivo con benznidazol por 30 días. No hubo nuevos hemocultivos positivos.
Altclas, *et al*., 1999 ^14^	1 paciente con leucemia mieloide crónica sometido a trasplante de médula ósea alogénico Strout semanal hasta superar la fase de inmunosupresión farmacológica	Paciente asintomático con tripomastigotes en sangre periférica en el día 101 del trasplante	Tratamiento preventivo con benznidazol por 7 semanas Desaparición de la parasitemia en el día 9 de iniciado el tratamiento
Altclas, *et al*., 2005 ^15^	1.328 pacientes sometidos a trasplante de médula ósea 25 con enfermedad de Chagas indeterminada sometidos a Strout, ELISA, inmunofluorescencia indirecta, hemaglutinación semanal durante dos meses, luego cada 15 días por un mes y luego mensual	1 paciente asintomático con linfoma no Hodgkin y hemocultivo positivo 1 paciente asintomáticocon linfoma no Hodgkin y Strout positivo 4 pacientes sometidos a trasplante alogénico de médula ósea Strout positivo en los días 16, 101, 120 y 178 del trasplante Un paciente con lesiones cutáneas por T. cruzi; los demás asintomáticos	Tratamiento preventivo con benznidazol por 30 días. No hubo nuevos hemocultivos positivos. Tratamiento preventivo con benznidazol por 20 días. Remisión de la parasitemia Tratamiento con benznidazol por 30 días. Strout semanal con normalización a los 14 días en todos los pacientes
Pinazo, *et al*., 2013 ^9^	5 pacientes con enfermedad de Chagas, 4 con neoplasia maligna hematológica Diagnóstico mediante PCR para T. cruzi	Una paciente asintomática con PCR positiva Dos con PCR negativa	Tratamiento con benznidazol y desaparición en la PCR a los 14 días Tratamiento con benznidazol
Chalela, *et al*., 2021 ^16^	1 paciente con linfoma Hodgkin sometido a trasplante de médula ósea	Strout positivo en el día 17 de neutropenia febril 56 días después de la primera terapia, nuevo síndrome febril con Strout positivo	Nifurtimox por 60 días Strout negativo a las tres semanas Benznidazol por 60 días Ausencia de recurrencia

Por otra parte, Altclas, *et al*., describieron un paciente de 27 años con leucemia mieloide crónica sometido a trasplante de médula ósea alogénico. A diferencia del corto seguimiento en el trasplante autólogo, en este caso se documentó *T. cruzi* en sangre periférica con el método Strout en el día 101 después del trasplante ^14^. No hubo síntomas clínicos y se administró el tratamiento preventivo con benznidazol durante siete semanas. A los nueve días de iniciado, la parasitemia era negativa.

En otro estudio en el 2005 en Argentina, Atclas, *et al*., describieron una serie de 25 pacientes con serología positiva para la enfermedad de Chagas sometidos a trasplante de medula ósea ^15^. Después del trasplante, se hizo el seguimiento de los pacientes usando el método de Strout directo y pruebas serológicas convencionales aplicadas semanalmente durante los primeros dos meses, cada 15 días durante el tercer mes y, después, mensualmente. El tiempo de seguimiento estipulado para el trasplante autólogo fue de 60 días y, para el alogénico, todo el periodo de inmunosupresión. Cuatro pacientes con trasplante autólogo y dos con trasplante alogénico presentaron recurrencias detectadas mediante el método de Strout. Un paciente tuvo lesiones cutáneas. Se prescribió tratamiento con benznidazol durante 4 a 8 semanas como terapia preventiva, consiguiéndose el aclaramiento de la parasitemia.

Por otra parte, la estrategia descrita por Dictar, *et al*., permitió la detección temprana de la parasitemia y la instauración de la terapia efectiva. No se reportó seguimiento después de los dos meses del trasplante ^17^, sin embargo, se abarcó el periodo de mayor propensión a la reactivación de la enfermedad. Al igual que en el estudio de Altclas, *et al*., no se reportaron efectos adversos asociados con el uso de benznidazol en mayo de 2022 (neuropatía periférica, erupción, agranulocitosis, etc.) y el seguimiento a dos años no evidenció reactivación de la enfermedad.

Pinazo, *et al*., por su parte, describieron una serie de cinco pacientes latinoamericanos (cuatro bolivianos y un argentino) con enfermedad neoplásica subyacente y enfermedad de Chagas ^9^. Dos de ellos tenían leucemia mieloide aguda, uno, mieloma múltiple y, una paciente, adenocarcinoma de mama. A una mujer de 44 años se le había diagnosticado enfermedad de Chagas en el 2005 en una forma clínica indeterminada y, en el 2010, se le diagnosticó mieloma múltiple IgG kappa. Durante 12 meses se le hizo seguimiento mediante PCR para *T. cruzi*. Una vez salió positiva, recibió el tratamiento preventivo con benznidazol, con lo cual la PCR fue negativa a los 14 días. La paciente no presentó manifestaciones clínicas y en el seguimiento anual no había presentado recaídas.

Durante la reactivación, las manifestaciones clínicas incluyen síndrome febril agudo, miocarditis, meningoencefalitis y lesiones cutáneas ^7^^,^^10^. Chalela, *et al*., recientemente describieron el caso de un hombre colombiano de 62 años con diagnóstico de linfoma de Hodgkin clásico, quien había recibido quimioterapia de alta intensidad como esquema terapéutico condicionante antes del trasplante autólogo y después de una primera recaída ^16^. En la tamización se documentó enfermedad de Chagas. Tres días antes del trasplante, presentó fiebre y elevación de la proteína C reactiva, sin evidencia clínica de infección y con hemocultivos negativos, por lo que se inició el manejo con un carbapenémico; a los dos días del trasplante, presentó nuevamente fiebre, pero no se documentó un foco infeccioso. En el día 17 después del trasplante, la fiebre persistía. No hubo alteraciones cutáneas, miocárdicas, gastrointestinales ni neurológicas. En ese momento, se hizo un micro Strout y se evidenciaron tripomastigotes. Se inició su manejo con nifurtimox durante 60 días. A las tres semanas, el micro Strout se tornó negativo; sin embargo, en el día 54 de finalizado el tratamiento presentó síndrome febril nuevamente y se detectaron tripomastigotes en el micro Strout. Entonces, se administró benznidazol durante 60 días, con lo que no hubo otras reactivaciones.

Estas reactivaciones pueden verse también en pacientes con inmunosupresión celular, como aquellos con infección por HIV. En el caso publicado por Diazgranados, *et al*., una mujer de 26 años asistió al seguimiento ambulatorio de su infección por VIH en julio del 2003, después de una interrupción de 14 meses; en mayo del 2022, el conteo de células CD4 fue de 189 por microlitro y, la carga viral, de 222.000 copias/ml; además, la paciente refirió cefalea progresiva y debilidad del hemicuerpo izquierdo. En el examen físico, tenía fiebre de 38,5 °C y hemiparesia izquierda. En la tomografía axial computarizada de cráneo, se detectó una lesión que ocupaba espacio. Dados sus antecedentes serológicos, se consideró una toxoplasmosis cerebral y se inició el tratamiento con clindamicina más pirimetamina, pero cinco semanas después la cefalea empeoró, la paciente no podía caminar y presentaba convulsiones. Fue hospitalizada y se le hizo la punción lumbar que evidenció pleocitosis linfocítica leve (23 leucocitos, 60 % linfocitos y 40 % mononucleares), hipoglucorraquia (22 mg/dl) e hiperproteinorraquia (320 mg/dl), así como parásitos flagelados correspondientes a tripomastigotes. En la biopsia cerebral, se encontró inflamación aguda y crónica, necrosis, hemorragia y gliosis astrocítica, pero no se vieron formas parasitarias. La serología por inmunofluorescencia indirecta de IgG fue positiva para *T. cruzi*.

Como se ha descrito, la evidencia clínica actual de reactivación de la enfermedad de Chagas se recoge en series y reportes de casos, y no presenta variaciones significativas en las últimas dos décadas. En el caso del trasplante autólogo, persiste la recomendación de hacer tamización con serología previa al trasplante ^12^. A partir de las experiencias aquí descritas, en caso de positividad, la valoración semanal clínica y paraclínica permite la detección temprana de la parasitemia y su intervención terapéutica, lo que parece ser una estrategia que previene la aparición de complicaciones sistémicas. En este sentido, la prueba diagnóstica que se utilice dependerá de la disponibilidad local, los recursos y la experiencia, pero se prefieren la prueba de Strout y la PCR. En caso de documentarse parasitemia, los reportes recomiendan el uso de benznidazol sobre el nifurtimox, dados los efectos adversos, interacciones farmacológicas y descripciones de recurrencia reportadas con este último.

Hasta donde se sabe, no hay información publicada sobre el uso de medicamentos para la profilaxis en pacientes con enfermedad de Chagas crónica sometidos a trasplante autólogo. Se requieren más estudios para determinar herramientas que permitan estimar la probabilidad de reactivación de la enfermedad y establecer la mejor relación de costo, riesgo y beneficio de la terapia profiláctica. En países endémicos para la enfermedad de Chagas, es imprescindible incluir la serología para detectarla antes del trasplante; un resultado positivo necesariamente requiere descartar su reactivación en los casos de neutropenia febril.
